# Large Congenital Mesenteric Defect Presenting in an Adult

**DOI:** 10.4103/1319-3767.65193

**Published:** 2010-07

**Authors:** Zia ur Rehman, Sadaf Khan

**Affiliations:** Department of Surgery, Aga Khan University Hospital, Karachi, Pakistan

**Keywords:** Internal hernias, mesenteric defect, Intestinal obstruction, bowel ischemia

## Abstract

Congenital internal hernia is a rare cause of bowel obstruction in adults and often presents with complications. A high index of suspicion, occasionally aided by appropriate radiological imaging, should lead to early surgical intervention and thus reduce morbidity and mortality. We describe a case of a 27-year-old woman who presented with upper abdominal pain and nonspecific abdominal signs. Computed tomography showed features of bowel ischemia which prompted surgical intervention. On exploration, she was found to have a large mesenteric defect with herniating ileum and ascending colon. A segment of gangrenous small bowel was resected. The mesenteric defect was repaired and the bowel tacked down to prevent volvulus. The patient made an uneventful recovery.

Internal hernias are a rare cause of small bowel obstruction.[1] Transmesenteric hernia is a type of internal hernia.[2] These mesenteric defects can be congenital or acquired. Patients can present with intestinal obstruction at any age. Most of the documented cases of congenital mesenteric defects as a cause of internal hernias are described in the pediatric population. We present a case of a previously asymptomatic 27-year-old woman with a large mesenteric defect involving almost the entire small bowel mesentery. As far as we are aware, such a large mesenteric defect has not been reported previously.

## CASE REPORT

**Figure 1:**
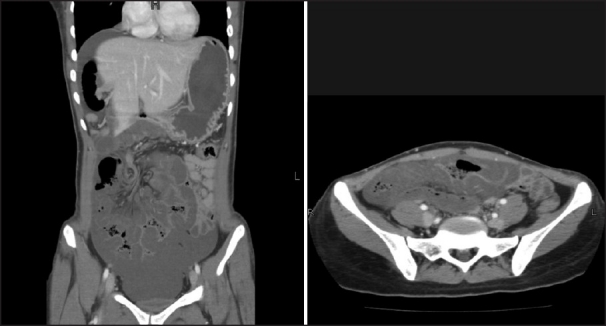
CT scan images showing dilated small bowel loops, significant free fluid and intramural air

**Figure 2:**
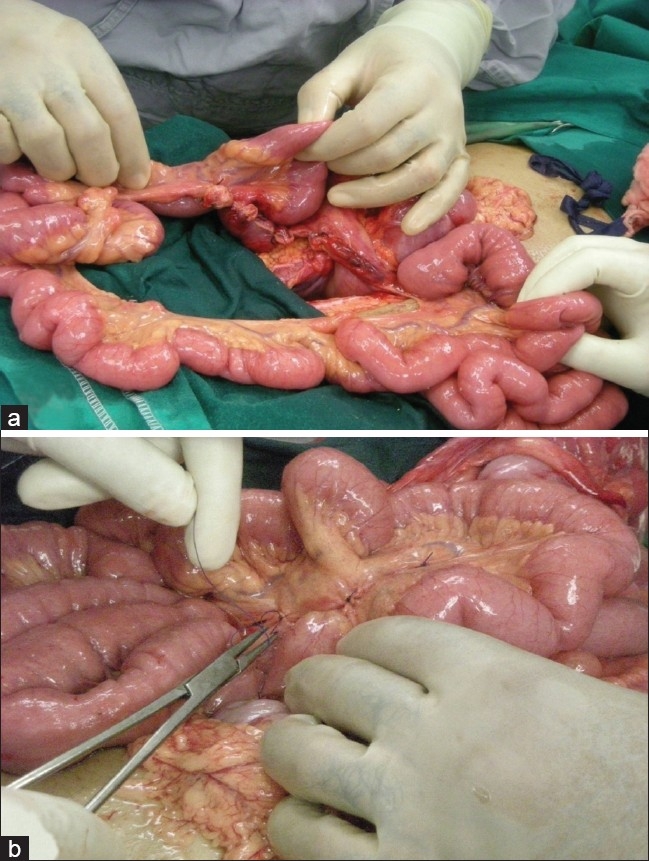
(a) Large mesenteric defect through which small bowel and cecum were herniating; (b) defect being closed primarily

**Figure 3:**
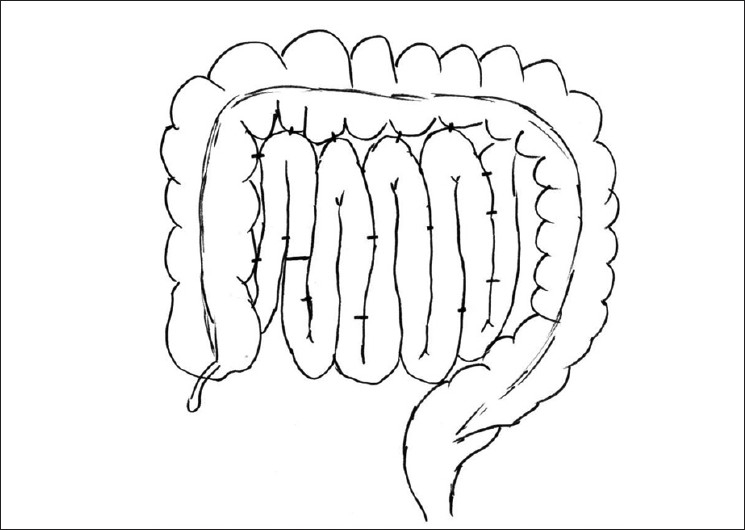
Final configuration of small bowel loops after closure of large mesenteric defect to make broad mesenteric base, which is unlikely to twist

An otherwise healthy 27-year-old woman presented with severe upper abdominal pain, multiple episodes of vomiting and absolute constipation for the previous 12 hours. The pain was acute in onset, severe and continuous. It was initially located in the epigastrium but became generalized with progression of time. It was severe enough to warrant a continuous intravenous infusion of meperidine. The pain was associated with multiple episodes of nonbilious vomiting. She had no other comorbid conditions and had not undergone any operations in the past. On examination, she was drowsy with a pulse of 110/minute, blood pressure of 90/50 mm Hg, temperature of 37°C and respiratory rate of 23/minute. She was dehydrated. Abdominal examination revealed diffuse abdominal tenderness and voluntary guarding in the right lower quadrant. Bowel sounds were audible. A reducible paraumbilical hernia with a 2 × 2 cm fascial defect was also noticed. Breath sounds in both lungs were equal and vesicular. Results of laboratory investigations were normal except for a borderline elevated white blood cell count [Table 1]. A CT scan of the abdomen was done. This revealed dilated distal small bowel with features of bowel ischemia and significant free fluid [Figure 1]. After initial resuscitation, the abdomen was explored through a midline incision. Approximately 1.5 liters of reactionary peritoneal fluid was drained. The proximal ascending colon and two thirds of small intestine had herniated through a large mesenteric defect. Fifteen centimeters of mid-ileum was grayish-black in color with loss of peristalsis and absent pulsations. The terminal ileum was viable. Pulsations of mesenteric vessels were intact. On splaying out the bowel, a large mesenteric defect measuring approximately 12 × 15 cm was noted. The arcades in the mesentery were intact running in close proximity to the mesenteric edge of the bowel. Distal to the proximal jejunum, true mesentery was absent. There was no evidence of malrotation. A redundant sigmoid colon was also appreciated. The gangrenous segment of small bowel was resected and a stapled side-to-side functional anastomosis created. The previously described mesenteric defect was closed. This resulted in a configuration that would have a very high likelihood of twisting. To minimize this risk, the small bowel loops were systematically positioned side by side. The arrangement was secured by intermittent tacking sero-muscular sutures placed between bowel loops. Care was taken to avoid kinking of the small bowel [Figures 2, 3]. The patient tolerated the procedure well. Bowel function resumed on the third postoperative day. Diet was initiated and tolerated. She was discharged on the sixth postoperative day, the delay being due to social issues. On follow-up visit at 2 months, she was doing well.

**Table 1:** Laboratory parameters

Laboratory parameter	Result
Hemoglobin	11 g/dL
WBCs	11600/cc
BUN	11 mg/dL
S. Creatinine	0.9 mg/dL
S. Sodium	132 mEq/dL
S. Potassium	3.2 mEq/dL
S. Amylase	70 IU/dL

## DISCUSSION

In about 0.2% to 0.9% of cases of small bowel obstruction, the obstruction is due to internal herniation. Herniation can be congenital or acquired. Common sites of internal hernias are paraduodenal (50%), supra- and/ or peri-vesical, intersigmoid, foramen of Winslow, omentum, postoperative mesenteric defects and congenital mesenteric defects.[3,4]

Mesenteric defects are an established cause of internal herniation in nonoperated abdomens and provide a potential site for intestinal incarceration or strangulation. Congenital mesenteric defects most often occur in the small bowel mesentery and less commonly in the colonic mesentery. The vast majority of these cases have been reported in infants or children, often with an associated intra-abdominal anomaly. Murphy found that of the 11 infants presenting with herniation through a mesenteric defect of the small intestine, 10 had an associated anomaly, the most common being intestinal atresia.[5] In adults, defects are most commonly acquired as a result of either blunt abdominal trauma or surgical manipulation of the bowel and mesentery. However, in the case we are reporting, the mesenteric defect was congenital and not associated with an intestinal anomaly.

The most common location of mesenteric defects is in the region of the small bowel (70% of cases), with 53% of these being in the ileocecal area of the mesentery.[6] These defects are typically small, although there are rare reports of large defects, as seen in our patient.[7]

Mesenteric defects present with a spectrum of clinical features — from being asymptomatic to being a cause of unexpected death. Byaud autopsied two patients for unexpected death, one child of 3 days and a 23-year-old woman, and found bowel herniation through a congenital mesenteric defect to be the cause of death.[8]

A high index of suspicion for congenital mesenteric defects is warranted in patients who present with features of intestinal obstruction in the absence of obvious external hernia or previous abdominal surgery. Operative management consists of timely laparotomy, reduction of hernia, resection/ anastomosis of devitalized bowel and closure of the defect.[2] The hernia ring may require manual dilatation or enlargement to assist in reduction of the hernia. The mesenteric defect or rent should always be closed. A defect near the root of mesentery may pose a challenge to closure due to limited exposure. Care must be taken to preserve mesenteric vessels near the edge of the defect. In this patient, closure of the defect resulted in a mesentery with a narrow root, susceptible to volvulizing. To minimize this risk, we used a technique utilized for the management of pediatric patients with recurrent idiopathic intussusceptions. This involves laying the small bowel along the length of the ascending colon and tacking it in place with sero-muscular stitches. In addition to the ascending colon, we also placed the small bowel side by side in a sequential manner and tacked the loops in position [Figure 3]. This maneuver broadened the root of the mesentery, and we presume, reduced the risk of volvulus.

## CONCLUSION

Severe unexplained abdominal pain in adults can be due to transmesenteric hernia. Diagnosis requires high index of suspicion, urgent surgical exploration and correction of the mesenteric defect. The innovation described is suggested for the management of patients with large mesenteric defects.
